# A Rare Case of Pulmonary Edema Secondary to Hydrochlorothiazide Use

**DOI:** 10.1155/2024/9423545

**Published:** 2024-11-07

**Authors:** Evan J. Chen, Laurie Hayrapetian, Katherine Frishe

**Affiliations:** ^1^Department of Internal Medicine, Los Angeles General Medical Center, Los Angeles, California, USA; ^2^Department of Internal Medicine, Keck Hospital of USC, Los Angeles, California, USA

**Keywords:** hydrochlorothiazide, idiosyncratic drug reaction, medication side effects, noncardiogenic pulmonary edema, thiazide diuretics

## Abstract

Noncardiogenic pulmonary edema has been reported as a rare adverse reaction of hydrochlorothiazide. Symptoms can develop acutely after medication ingestion, and patients may present acutely ill. The mechanism by which hydrochlorothiazide causes pulmonary edema remains unknown and is considered idiosyncratic. Prompt supportive care and discontinuation of the medication is necessary to prevent and manage such a complication. This case report describes a patient who developed noncardiogenic pulmonary edema after taking a combination pill of hydrochlorothiazide-losartan.

## 1. Introduction

Noncardiogenic pulmonary edema (NCPE) has been reported as a rare but life-threatening adverse reaction of hydrochlorothiazide (HCTZ). HCTZ has been widely used for the management of hypertension globally due to its relatively safe profile. The most common adverse effects of HCTZ include orthostatic hypotension, electrolyte abnormalities (hyponatremia, hypokalemia, hypercalcemia, and hypomagnesemia), hyperglycemia, hyperuricemia, and hyperlipidemia. Few cases of HCTZ-induced NCPE have been observed and described [1–8]. Here, we report a case of a patient presenting with acute hypoxemic respiratory failure and pulmonary edema following ingestion of HCTZ.

## 2. Case Presentation

**Figure 1:**
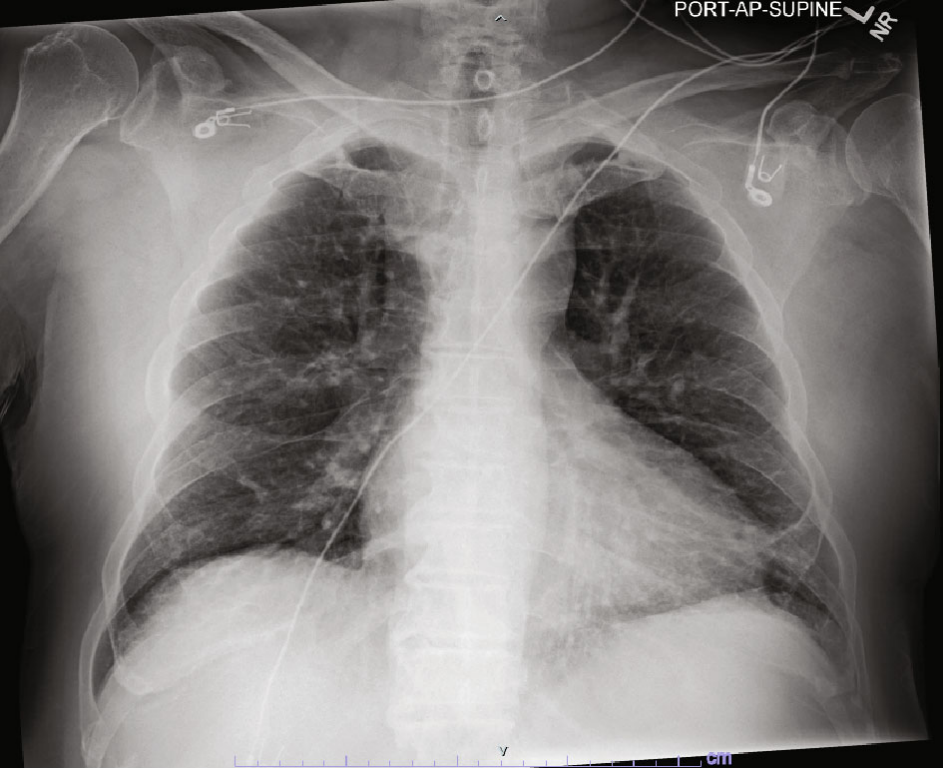
Chest x-ray demonstrating bilateral interstitial opacities concerning for pulmonary edema.

A 73-year-old male with a past medical history of hypertension and benign prostatic hyperplasia presented to the emergency department with acute onset generalized weakness, nausea, emesis, diarrhea, fever, and dyspnea approximately 2.5 h after taking a combination pill of HCTZ-losartan. The patient reported that he had been prescribed losartan-HCTZ 50-12.5 mg 7 months earlier but had self-discontinued the medication due to the onset of mouth sores and generalized weakness after taking the combination pill. He subsequently returned to his clinic in hypertensive urgency and was counseled to resume the combination pill. Upon resumption, he developed his current symptoms and presented to the emergency department. His vital signs on presentation were notable for a temperature of 39.3°C, blood pressure of 137/66 mmHg, heart rate of 131 beats per minute, respiratory rate of 33 breaths per minute, and an oxygen saturation of 88% on room air. Physical exam was notable for the presence of bilateral lung crackles and diaphoresis. Point-of-care ultrasound revealed B-lines in bilateral lung fields and a normal ejection fraction. Electrocardiogram showed sinus tachycardia. Chest x-ray showed mild bilateral interstitial opacities (Figure 1). Labs were notable for leukopenia (white blood cell count 2.0 K/cubic millimeter (cumm), with baseline 5.7 K/cumm) and elevated procalcitonin (58.70 ng/mL), and an unremarkable comprehensive metabolic panel, respiratory viral panel, troponin, and probrain natriuretic peptide. The patient was placed on nasal cannula with improvement in oxygenation. He was admitted to the hospital for acute hypoxic respiratory failure and started on antibiotics for possible community-acquired pneumonia. The patient's tachycardia, tachypnea, fever, pulmonary crackles, and oxygen requirement resolved within 5 h of presentation, and he was discharged the following morning on losartan only for his hypertension. He was counseled to discontinue HCTZ indefinitely.

On primary care clinic follow-up, the patient reported no recurrence of symptoms with discontinuation of HCTZ. He reported compliance to losartan without any side effects.

## 3. Discussion/Conclusion

NCPE may have life-threatening clinical presentations and require prompt supportive care and management of the underlying etiology, which typically involves sepsis, disseminated intravascular coagulation, trauma, pancreatitis, blood transfusions, or recreational drug use. Several cases of HCTZ-induced NCPE have been observed and described in the literature [1–8]. Symptoms develop within minutes to hours of ingestion and can occur on first exposure to the medication or with intermittent use [1–3]. Symptoms include dyspnea, wheezing, and cough (90%) and, less frequently, fever, chills, and gastrointestinal symptoms such as nausea, vomiting, and diarrhea [1]. Symptoms of anaphylaxis such as hypotension and urticaria or flushing are notably absent. Patients may initially present with acute clinical deterioration, including hypotension and hypoxemia, requiring the use of vasopressors and/or noninvasive or invasive ventilation. Symptoms usually resolve within 3 days [1].

The pathophysiologic mechanism by which HCTZ causes pulmonary edema remains unknown and is considered idiosyncratic [1, 2, 4, 5]. Labs may show transient peripheral leukopenia secondary to intrapulmonary sequestration of neutrophils seen on bronchoalveolar lavage, which may indicate that HCTZ-induced lung injury is neutrophil-mediated [4, 6]. Labs may also show evidence of hemoconcentration and an elevated procalcitonin level, the cause of which is unknown [1, 4, 6]. Eosinophilia is notably absent [6]. A HCTZ rechallenge test for diagnosis is deemed impractical due to the potential severity of the reaction. Cross-reactivity does not appear to occur with loop diuretic use, and symptoms do not appear to be dose-dependent [7]. Management involves the discontinuation of the offending medication as well as supportive care. Though corticosteroids have been utilized in certain cases, there is currently no evidence that these medications may reduce the frequency or severity of HCTZ-induced NCPE [8].

Acute NCPE is a rare but life-threatening clinical condition that must be recognized and managed promptly. Our patient's case had many features consistent with previous reports of HCTZ-associated pulmonary edema, including fever, hypoxia, leukopenia, and an elevated procalcitonin level. It also had features not previously described, namely, the outbreak of mouth sores with previous use of HCTZ. This case highlights the importance of considering HCTZ as a cause of NCPE, as NCPE requires prompt supportive care. Patients should be counseled to discontinue thiazide diuretics indefinitely.

## Data Availability

Data sharing is not applicable to this article as no new data were created or analyzed in this study.

## References

[1] Traversa M., Collini A., Villois P., Elia F., Verhovez A., Aprà F. (2018). When a diuretic causes pulmonary oedema. *European Journal of Case Reports in Internal Medicine*.

[2] Kavaru M. S., Ahmad M., Amirthalingam K. N. (1990). Hydrochlorothiazide-induced acute pulmonary edema. *Cleveland Clinic Journal of Medicine*.

[3] Knowles S. R., Wong G. A., Rahim S. A., Binkley K., Phillips E. J., Shear N. H. (2005). Hydrochlorothiazide-induced noncardiogenic pulmonary edema: an underrecognized yet serious adverse drug reaction. *Pharmacotherapy*.

[4] Darwish O. S., Criley J. (2011). Hydrochlorothiazide-induced noncardiogenic pulmonary edema: BAL fluid analysis. *Chest*.

[5] Bell R. T., Lippmann M. (1979). Hydrochlorothiazide-induced pulmonary edema: report of a case and review of the literature. *Archives of Internal Medicine*.

[6] Bernal C., Patarca R. (1999). Hydrochlorothiazide-induced pulmonary edema and associated immunologic changes. *The Annals of Pharmacotherapy*.

[7] Fine S. R., Lodha A., Zoneraich S., Mollura J. L. (1995). Hydrochlorothiazide-induced acute pulmonary edema. *The Annals of Pharmacotherapy*.

[8] Jansson P. S., Leisten D. C., Sarkisian T. M., Wilcox S. R., Lee J. (2018). Recurrent hydrochlorothiazide-induced acute respiratory distress syndrome treated with extracorporeal membrane oxygenation. *The Journal of Emergency Medicine*.

[9] Chen E., Hayrapetian L., Frishe K. A Rare Case of Noncardiogenic Pulmonary Edema Secondary to Hydrochlorothiazide Use. Poster presentation.

